# Influence of alexithymia severity in the healthy population on the susceptibility to false interoceptive feedback

**DOI:** 10.3389/fpsyg.2024.1442138

**Published:** 2025-01-08

**Authors:** Hitomi Ikarashi, Naofumi Otsuru, Sena Takahashi, Kazuaki Nagasaka, Masayuki Hara, Hideaki Onishi

**Affiliations:** ^1^Department of Physical Therapy, Niigata University of Health and Welfare, Niigata, Japan; ^2^Institute for Human Movement and Medical Sciences, Niigata University of Health and Welfare, Niigata, Japan; ^3^Graduate School of Science and Engineering, Saitama University, Saitama, Japan

**Keywords:** alexithymia, virtual reality, embodiment, neuroscience, interoception, interoceptive feedback

## Abstract

Alexithymia is a psychological trait characterized by difficulty expressing emotions. Previous studies reported that individuals with higher alexithymia have a decreased sense of interoception, which is the sense of monitoring and controlling internal organs. Thus, we hypothesized that internal organ activity (cardiac activities in the present study) was easily affected by false feedback in individuals with severe alexithymia. Therefore, we investigated whether the effects of fake heart rate feedback on real cardiac activities differ depending on the severity of alexithymia as assessed by the 20-item Toronto Alexithymia Scale (TAS-20). Fake heart rate feedback was presented as if it were occurring in the individual’s hand through a virtual reality system at various speeds. Changes in cardiac activities were evaluated by the root-mean-square-successive difference (RMSSD, high value indicates greater parasympathetic tone) of the heartbeat interval. Our findings revealed a negative correlation between externally oriented thinking, a subscale of the TAS-20 score, and the RMSSD change ratio elicited by fake heart rate feedback. These findings indicate that individuals with higher alexithymia might be particularly susceptible to external fake feedback.

## 1 Introduction

Alexithymia, characterized by difficulty identifying and expressing emotions, was first described by Sifneos (1973). Its prevalence is estimated at around 10% in the general population (Salminen et al., 1999; Shibata et al., 2014) and rises to 40–60% among patients with psychosomatic illness (Taylor, 2000). Assessment of Alexithymia commonly employs the 20-item Toronto Alexithymia Scale (TAS-20), which includes three subscales: difficulty identifying feelings (DIF), difficulty describing feelings (DDF), and externally oriented thinking (EOT) (Bagby et al., 1994; Herbert et al., 2011). A previous study reported Cronbach’s *α* values of 0.79, 0.75, and 0.66 for TAS-20-DIF, TAS-20-DDF, and TAS-20-EOT, respectively (Bagby et al., 1994), highlighting their adequate internal reliability. Research indicates a correlation between higher levels of depression and alexithymia (subjects with a TAS-20 score > 61) (Honkalampi et al., 2000). Additionally, alexithymia is associated with anxiety disorders, autism (Berardis et al., 2008; Gaigg et al., 2018; Poquérusse et al., 2018), and chronic pain (Shibata et al., 2014). However, the physiological characteristics present in highly alexithymic individuals remain unclear.

Recently, the relationship between alexithymia and interoception has garnered attention. Interoception, defined as an individual’s perception of their body’s physiological condition through the processing of internal information by the nervous system, is thought to underpin emotion (Craig, 2002; Critchley and Garfinkel, 2017). Prior studies have reported that interoception dysfunction is implicated in alexithymia (Brewer et al., 2016; Murphy et al., 2018). One study demonstrated that a high TAS-20 score is associated with poor performance in the heartbeat-tracking task, an evaluation of interoceptive accuracy (Shah et al., 2016). This study suggested that high alexithymia is linked to a reduced ability to monitor one’s internal state, as evidenced by the fact that lesions of the insula, a key cortical region for interoceptive processing, were associated with acquired alexithymia (Hogeveen et al., 2016). Moreover, a higher TAS-20 score is associated with an increased risk of cardiovascular mortality, an association that remains significant even when controlled for age, smoking, alcohol consumption, and physical activity (Tolmunen et al., 2010). These studies suggest that alexithymia influences both the monitoring and modulation of one’s internal state.

In our study, we explored the relationship between alexithymia and the ability to monitor internal states. Specifically, we examined how real cardiac activity responded to false heart rate feedback in a virtual reality (VR) environment. A VR system was developed to visually present the false cardiac feedback on virtual hands through a head-mounted display (HMD). VR is a technology in which participants are immersed in a computer-generated virtual environment, allowing them to experience simulations of sensations and behaviors that differ from reality. The use of VR might provide participants with greater immersion than conventional feedback, potentially resulting in greater benefits. Research has demonstrated that VR increased attention to heart rate variability biofeedback (Rockstroh et al., 2019). Moreover, research using acoustic feedback found that heart rates were not significantly modulated (Iodice et al., 2019). Therefore, we chose VR for false heart rate feedback. We hypothesized that subjects with higher TAS-20 scores, indicating a greater tendency toward alexithymia, would have their real cardiac activity modulated by false cardiac feedback through VR due to their reduced accuracy in monitoring their internal states.

## 2 Materials and methods

### 2.1 Participants

We recruited 42 healthy participants (20 males and 22 females; average age 20.67 ± 0.47 years) from Niigata University of Health and Welfare. These participants were not on any medication and did not have any neurological or psychiatric disorders. They were randomly divided into three groups of 14 participants each, based on the deviation of false heart rate feedback: no-change condition, low-increase condition, or high-increase condition (details are described in the item for “heartbeat feedback” by VR). The study was conducted in accordance with the Declaration of Helsinki and was approved by the ethics committee of Niigata University of Health and Welfare (18661–210,625). All participants provided written informed consent before the experiment.

### 2.2 Assessment for alexithymia

The TAS-20, a widely accepted measure for alexithymia, consists of 20 items rated on a 5-point scale, with a total score of 100 points (Bagby et al., 1994). It includes three subscales: DIF (TAS-20-DIF; scores: 0–35), difficulty describing feelings to others (TAS-20-DDF; scores: 0–25), and an externally oriented style of thinking (TAS-20-EOT; scores: 0–40). Participants completed the Japanese version of the TAS-20, which has demonstrated good internal consistency (Cronbach’s *α* of 0.85, 0.72, and 0.58 for the TAS-20-DIF, TAS-20-DDF, and TAS-20-EOT, respectively) (Gen et al., 2003).

### 2.3 Experimental setup

We developed a virtual reality system to investigate whether real cardiac activity in participants prone to alexithymia is significantly influenced by false cardiac feedback. The system provided visual false heartbeat feedback via virtual hands. The virtual environment was created using Thorn et al. (2019) (Unity Technologies, United States) and displayed to participants on a HMD (Oculus Rift S, Oculus, United States). Hand and finger positions were tracked using a leap motion device (Leap Motion, UK) with Leap Motion Orion 3.2.1 (SDK). The virtual hands were rendered in Leap Motion Core Asset 2.3.1 for Unity. In addition to realistic virtual hands, ghost hands with identical shapes but red skin were also rendered. The artificial heartbeat was expressed by modulating the alpha value of the ghost hands over time.

### 2.4 Experimental procedures

**Figure 1:**
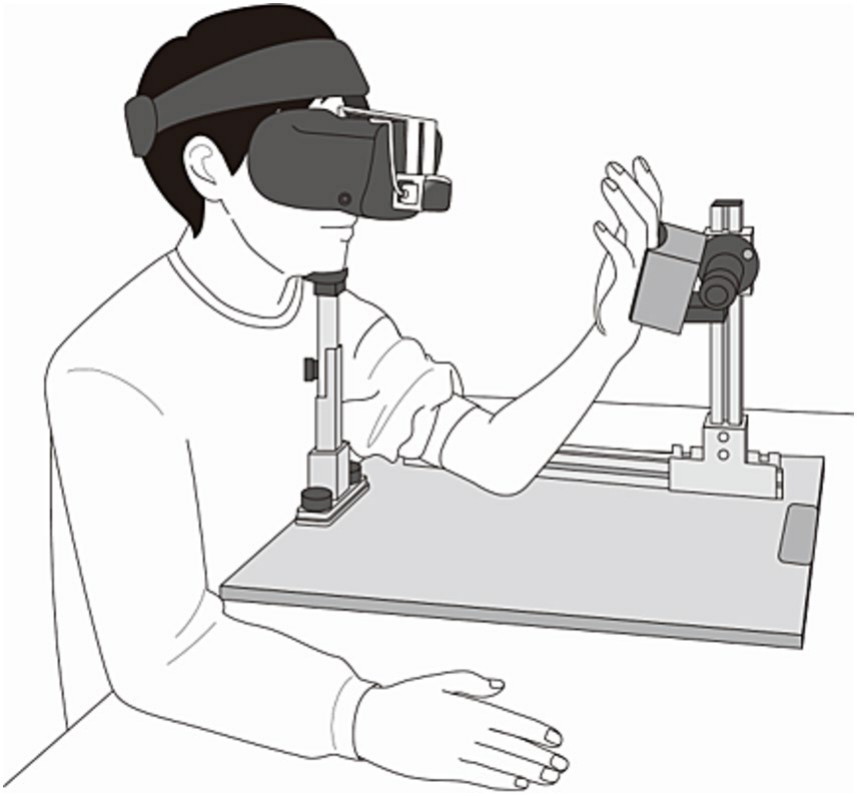
Positions of the participant’s head and hand during the experiment. The direct distance between the head-mounted display (HMD) and the palm surface is ~200 mm. The head and hand positions are stabilized using a chin rest and an armrest, respectively.

**Figure 2:**
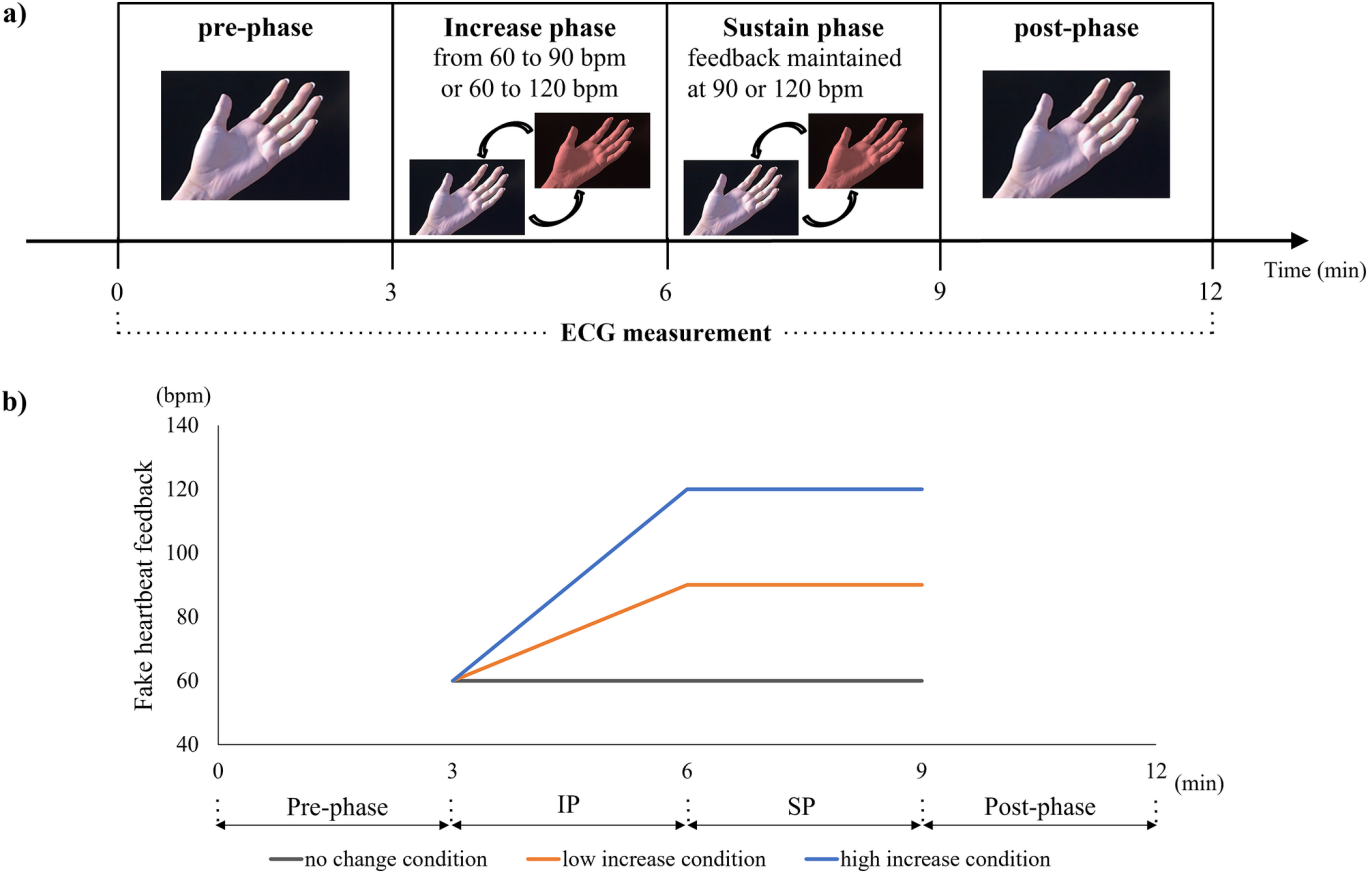
**(A)** Experimental procedure. The experiment consists of four phases: pre-phase, increase phase (IP), sustain phase (SP), and post-phase. Each phase lasts for 3 min, and cardiac responses are measured using an electrocardiogram for all phases. **(B)** The pattern of fake heartbeat feedback from pre-phase to post-phase. In the no-change condition, a red flashing signal is consistently displayed to the virtual hand at a fixed rate of 60 beats per minute (bpm) throughout IP and SP. In the low-increase condition, the flashing feedback gradually increases linearly from 60 to 90 bpm during IP and remains constant at 90 bpm during SP. In the high-increase condition, the feedback progressively increases from 60 to 120 bpm during IP and remains at 120 bpm throughout SP.

Participants, while seated, placed their chin on a custom-made chin rest to limit head movement during the experiment. The participant’s left hand was positioned on a custom-made hand rest to maintain a consistent distance from the HMD, adjusted to approximately 200 mm (Figure 1). Figure 2A outlines the experiment’s paradigm. Initially, only a left-handed model was displayed on the HMD for three minutes (pre-phase). During the increase phase (IP) and sustain phase (SP), each lasting three minutes, the hand on the HMD was superimposed with a red flashing signal representing false heartbeat feedback. Pace of this signal varied among participants in each group: no change, low increase for providing fast heart rate feedback within the normal range, and high increase for providing fast heart rate feedback that deviates from the normal range. We defined the normal heart rate as 60–90 bpm based on a previous study (Page et al., 2016; Somers et al., 1993). In the no-change condition, the red flashing signal maintained a constant pace of 60 beats per minute (bpm) during both the IP and SP. In the low-increase condition, the feedback linearly increased from 60 to 90 bpm during the IP and remained at 90 bpm during the SP. In the high-increase condition, the feedback escalated from 60 to 120 bpm during the IP and sustained at 120 bpm during the SP (Figure 2B). Thus, the low-increase condition provided mild deviation feedback (90 bpm), while the high-increase condition provided strong deviation feedback (120 bpm). In each condition, after the SP, only the hand image without feedback was displayed again for 3 min (post-phase). Prior to the experiment, participants were instructed: “Your heartbeat will be represented as a red flashing signal on your hand. Please keep your attention on your hand and refrain from moving during the experiment”.

### 2.5 Measurements of real activity during fake cardiac feedback

The participants’ cardiac responses were recorded using an electrocardiogram (ECG) device (FA-DL-310, 4Assist, Japan) throughout the experiment. We utilized ECG analysis software (Kubios HRV Premium, Finland) to compute the root-mean-square-successive difference (RMSSD) of the heartbeat interval difference during the pre-phase, IP, SP, and post-phase. RMSSD is a well-established indicator of parasympathetic function, with higher values indicating a greater parasympathetic tone (Nussinovitch et al., 2011). To assess the effects of fake feedback on cardiac control, we evaluated changes in RMSSD using the RMSSD change ratio, calculated by dividing the RMSSD values in the IP, SP, and post-phase by those in the pre-phase.

### 2.6 Statistical analysis

We employed the Kruskal–Wallis test, a nonparametric alternative to one-way ANOVA, to ensure that the RMSSD values during the pre-phase and TAS-20 subscale scores did not significantly differ among the conditions. To investigate the influence of external fake cardiac feedback on RMSSD, we conducted a mixed-design two-way ANOVA with fake feedback condition (no-change condition, low-increase condition, and high-increase condition) as interindividual factors and time (pre-phase, IP, SP, post-phase) as intra-individual factors. Furthermore, to explore whether higher levels of alexithymia are more susceptible to external fake cardiac feedback, we analyzed correlations between the RMSSD change ratio in the IP, SP, and post-phase and TAS-20 subscale scores. We utilized Pearson’s or Spearman’s correlation analyses, depending on the normality of the data determined using the Shapiro–Wilk test. For all statistical analyses, *p* < 0.05 was considered significant in the present study.

## 3 Results

First, we confirmed that there were no differences in the RMSSD during pre-phase and TAS-20 subscales among the participants across all conditions. The Kruskal–Wallis test revealed no significant difference between the DIF and DDF scores of participants across the conditions (*p* = 0.657 and *p* = 0.472 for the DIF and DDF scores, respectively). Similarly, the one-way ANOVA showed that the EOT score and pre-RMSSD did not differ among the conditions (*p* = 0.285 and *p* = 0.848 for the EOT score and RMSSD, respectively).

The results of the two-way ANOVA showed no significant interaction between the fake feedback conditions and time (F [2, 39] = 1.052, *p* = 0.390). Similarly, no significant main effects were observed (F [2, 39] = 0.138, *p* = 0.138).

**Figure 3:**
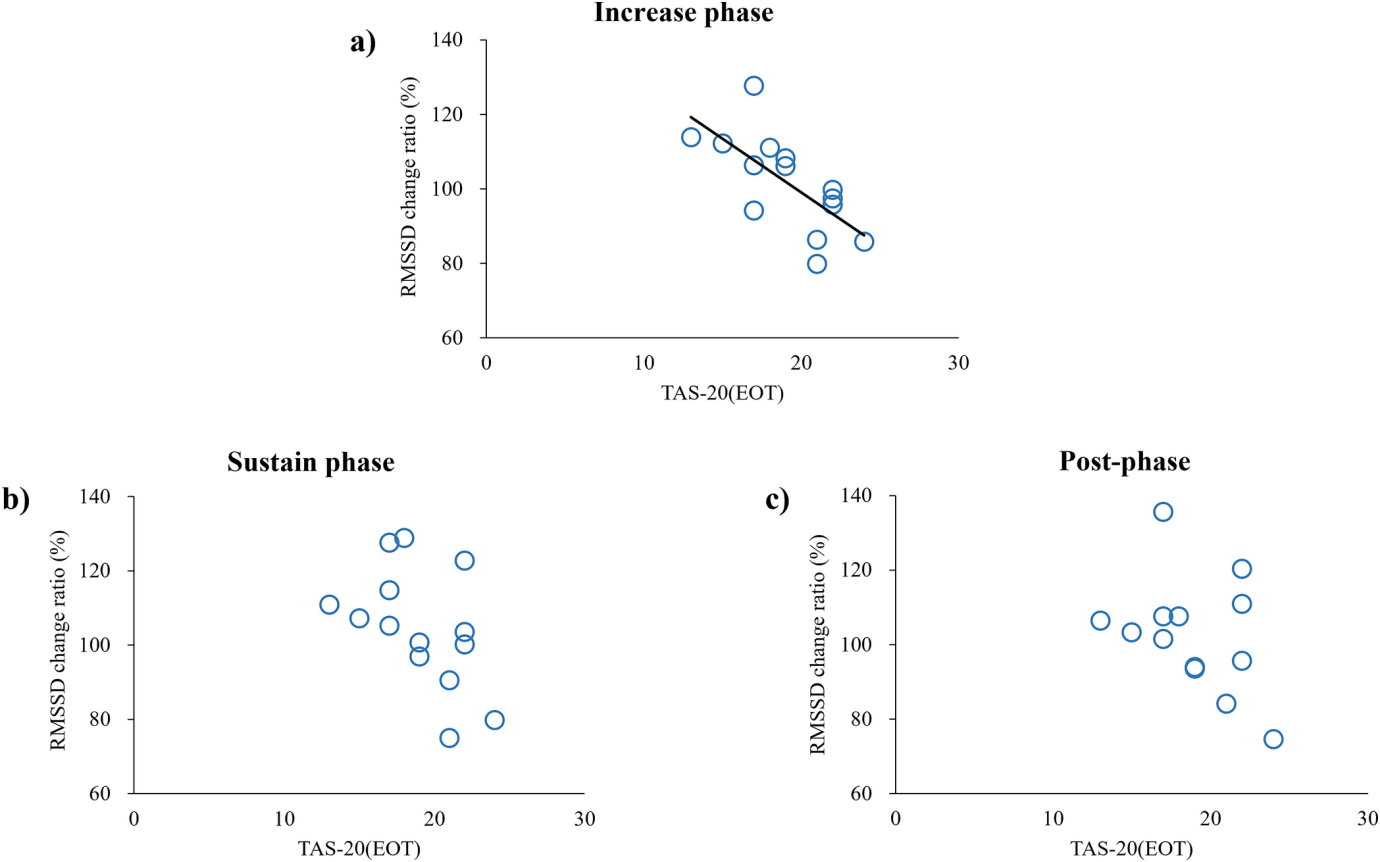
Correlation between RMSSD change ratio and TAS-20—an index of externally oriented thinking—in high-increase conditions. Each circle represents data obtained from each subject: **(A)** correlation in IP, **(B)** correlation in SP, and **(C)** correlation in the post-phase.

Table 1 presents the relationship between the RMSSD change ratio in the IP, SP, and post-phase and the TAS-20 subscale. Pearson’s correlation analysis showed a significantly negative correlation between the RMSSD change ratio and EOT during the IP in the high-increase conditions (*p* = 0.006, *r* = −0.69). A negative correlation was apparent in the SP, but it did not reach significance (*p* = 0.08, *r* = −0.48), and no significant correlation was observed in the post-phase (Figure 3). In contrast, no significant relationships were found between the RMSSD change ratio and the other TAS-20 subscales (DIF and DDF) in all phases. We confirmed that there was no significant relationship between the baseline RMSSD (pre-phase) and the subscale scores for TAS-20 across all conditions.

**Table 1:** Relationship between RMSSD change ratio and TAS-20 subscales.

		IP		SP		Post-rest post-phase	
		*r*	*p*-value	*r*	*p*-value	*r*	*p*-value
No change	DIF	0.09	0.77	−0.01	0.97	0.01	0.98
	DDF	0.04	0.89	−0.04	0.88	−0.05	0.86
	EOT	0.05	0.87	−0.06	0.83	−0.18	0.54
Low increase	DIF	0.22	0.44	0.30	0.30	0.54	0.05
	DDF	0.33	0.25	0.36	0.20	0.5	0.07
	EOT	0.26	0.37	0.19	0.53	0.18	0.55
High increase	DIF	−0.24	0.40	−0.43	0.13	−0.41	0.14
	DDF	0.07	0.82	−0.003	0.99	0.05	0.88
	EOT	−0.69	0.006*	−0.48	0.08	−0.41	0.15

TAS-20-DIF—the 20-item Toronto Alexithymia Scale (TAS-20) subscale regarding difficulty identifying feelings; TAS-20-DDF—TAS-20 subscale regarding difficulty describing feelings; TAS-20-EOT–TAS-20 subscale regarding externally oriented thinking. *; *p* < 0.05.

## 4 Discussion

In this study, we explored the relationship between alexithymia and cardiac response to false heart rate feedback using VR. Our results revealed a significant negative correlation between the RMSSD change ratio and EOT during the IP for the high-increase condition. This indicates that individuals with high levels of EOT exhibited a decrease in parasympathetic tone (evidenced by reduced RMSSD), indicating that their cardiac activities were following the false heart rate feedback, whereas individuals with low levels of EOT exhibited an increase in parasympathetic tone, indicating that they were resisting the false feedback. This supports our hypothesis that individuals with high levels of alexithymia are influenced by external false feedback due to their limited ability to monitor internal states. Previous studies have reported that a higher TAS-20 score is associated with poorer performance in heartbeat-tracking tasks (Shah et al., 2016) and that individuals with high levels of alexithymia show inaccuracies in respiration matching tasks (Abdulhamid et al., 2022). These findings suggest that individuals with severe alexithymia might have difficulties in accurately perceiving and regulating their internal states. Severe alexithymia is a risk factor for heart disease (Grabe et al., 2010; Tolmunen et al., 2010), and the results of the present study also suggest that autonomic nervous system regulation differs even under the same condition, depending on the level of alexithymia. Although the exact reason is unknown, it is interesting that people with high EOT scores controlled autonomic nervous system activity in a way that increased the heart load in the IP of fake feedback, whereas those with low EOT scores controlled autonomic nervous system activity in a way that decreased the heart load in the IP of fake feedback. We also observed a significant negative correlation between the RMSSD change ratio and EOT only during the IP for the high-increase condition. Results indicated that participants with high EOT have a different response to autonomic nervous system control when the deviation between the real heart rate and false feedback is sufficient. Regarding a significant relationship was observed with only EOT subscale, previous research showed a weak correlation between the EOT score and the DIF and DDF scores (Bagby et al., 1994). Similarly, in our study, we did not find any significant correlations between the EOT score and the DIF and DDF scores (data not shown). While the DIF and DDF assess the affective aspect of alexithymia, the EOT focuses on the cognitive aspect (Erni et al., 1997; Parker et al., 2003). Essentially, DIF and DDF involve verbalizing emotions based on internal information, whereas EOT relates to an excessive focus on the external world rather than internal states (Preece et al., 2017). In the present study, the participants were instructed as follows: “Your heartbeat will be represented as a red flashing signal on your hand. Please keep your attention on your hand.” Individuals with high EOT scores tend to focus on external stimuli and events opposed to internal states (Bagby et al., 1994). Thus, in subjects with high EOT, if there was a sufficient discrepancy between the actual heart rate and the feedback, RMSSD would have changed in the direction of sympathetic augmentation because of the high weighting of the external feedback. It has been reported that alexithymia is involved in dysfunction in the insular cortex, which controls the autonomic nervous system (Dobrushina et al., 2024; Hogeveen et al., 2016; Mantani et al., 2005). These previous findings supported the present results. Furthermore, the finding of a significant relationship only under the high-increase condition suggests that sufficient subjective awareness of false feedback is important for modulating RMSSD for subjects with high EOT.

Results of the present study need to be interpreted in light of several limitations. We only recruited young healthy volunteers, so it is unclear if similar relationships exist in populations with higher TAS-20 scores, such as patients with depression (Honkalampi et al., 2000). Future studies with patient populations are needed to address this issue. Additionally, we used fixed values for false heart rate feedback without considering individual differences in heart rate. This might have affected the change in RMSSD. However, when we examined the relationship between an individual’s baseline heart rate during pre-phase and the change in RMSSD caused by VR feedback, we found no significant relationship in either condition. Therefore, we believe that the effect of an individual’s baseline heart rate on the results of this study was limited. Moreover, it is undeniable that the effect might differ depending on the color used for feedback. In the present study, we used red color to evoke images of blood flow. Red was also used in research that provides heart rate feedback including VR (Suzuki et al., 2013; Sel et al., 2017). The effects of using other colors, such as cool colors, need to be verified in future research. Moreover, we do not fully understand the neural mechanisms responsible for the modulation of cardiac activity in response to fake feedback. Future research should investigate detailed neural mechanisms, such as the activities of the central autonomic network during fake feedback. In summary, our study provides new evidence suggesting that a higher EOT score is associated with reduced parasympathetic nerve control when given fake external heartbeat feedback contradicting the actual internal state. These findings support the notion that alexithymia would be linked to the monitoring and control of internal conditions.

## 5 Conclusion

This study aimed to elucidate the relationship between alexithymia and the ability to monitor one’s internal state. We examined the impact of false heart rate feedback, delivered via VR, on actual cardiac activity. The EOT subscale of the TAS-20 was found to correlate with cardiac responses when VR provided extreme false heart rate feedback. This suggests that individuals with high levels of alexithymia may have a low ability to monitor their internal states. Our findings provide insights into the relationship between alexithymia and interoception, aiding in the comprehension of pathophysiological mechanisms. These insights could also serve as a valuable biomarker for alexithymia.

## Data Availability

The original contributions presented in the study are included in the article, further inquiries can be directed to the corresponding author.
